# Invasive oral cancer stem cells display resistance to ionising radiation

**DOI:** 10.18632/oncotarget.6268

**Published:** 2015-11-02

**Authors:** Emilios Gemenetzidis, Luke Gammon, Adrian Biddle, Helena Emich, Ian C. Mackenzie

**Affiliations:** ^1^ Blizard Institute Barts and the London School of Medicine and Dentistry, Queen Mary University of London, London, UK

**Keywords:** irradiation, cancer stem cells, EMT, apoptosis, invasion

## Abstract

There is a significant amount of evidence to suggest that human tumors are driven and maintained by a sub-population of cells, known as cancer stem cells (CSC). In the case of head and neck cancer, such cells have been characterised by high expression levels of CD44 cell surface glycoprotein, while we have previously shown the presence of two diverse oral CSC populations *in vitro*, with different capacities for cell migration and proliferation. Here, we examined the response of oral CSC populations to ionising radiation (IR), a front-line measure for the treatment of head and neck tumors. We show that oral CSC initially display resistance to IR-induced growth arrest as well as relative apoptotic resistance. We propose that this is a result of preferential activation of the DNA damagerepair pathway in oral CSC with increased activation of ATM and BRCA1, elevated levels of DNA repair proteins RAD52, XLF, and a significantly faster rate of DNA double-strand-breaks clearance 24 hours following IR. By visually identifying CSC sub-populations undergoing EMT, we show that EMT-CSC represent the majority of invasive cells, and are more radio-resistant than any other population in re-constructed 3D tissues. We provide evidence that IR is not sufficient to eliminate CSC *in vitro*, and that sensitization of CD44^hi^/ESA^low^ cells to IR, followed by secondary EMT blockade, could be critical in order to reduce primary tumor recurrence, but more importantly to be able to eradicate cells capable of invasion and distant metastasis.

## INTRODUCTION

There has been an increasing amount of evidence in the recent years, suggesting that tumor populations with stem cell properties are responsible for recurrence after tumor therapy. Such populations have been described in both hematopoietic as well as in solid malignancies including breast, brain, pancreas, colon, and those of the head and neck [1–9]. Although their gene expression profiles are significantly different, all share common characteristics of stem cell behaviour. For example, they can replenish the tumor that they have originated from, as well as being able to reseed tumors when transplanted at limiting dilutions *in vivo*. CSCs are therefore regarded as being the most important target to achieve complete tumor eradication [10, 11].

In cancers of the head and neck, conventional irradiation is routinely used as means of therapy. Since the regulation of the DNA damage response (radio-sensitivity) depends on key checkpoint proteins, it is heavily influenced by cell cycle dynamics. As opposed to a single radiation dose, fractionation of the treatment ensures that cells will eventually be exposed in all different phases of the cell cycle during subsequent treatments. This is why ionising radiation treatment typically involves fractioned low-level radiation (usually 2Gy) over a period of time, to reach a final dose of up to 70Gy [12]. Ionizing radiation induces extensive DNA damage (mainly in the form of double strand breaks; DSBs) which initiates the pathway for genomic repair, or induces irreversible exit from the cell cycle that leads to cell death. Immediately after the induction of DSBs, the activation of ATM and ATR complexes act both as recruiters of additional substrates, as well as scaffolds for the assembly of additional active complexes [13]. ATM activates, via phosphorylation, additional downstream substrates including H2AX histone, an important regulator of the DNA damage response (DDR) mechanism that is recruited to sites of DSBs. In fact, phosphorylated H2AX (γH2A.X) has been extensively used not only as a marker of DNA damaged cells, but also as an indicator for the efficiency of repair following DNA damage.

Knowledge of the response of cancer stem cells to ionizing radiation is limited to only a few tumor types, but nonetheless suggests that CSCs are more radio-resistant than the remaining bulk of tumor cells. In breast tumor cell lines, stem cell-enriched mammospheres (CD24^low^/CD44^hi^) show greater clonogenic capacity and diminished numbers of γH2A.X foci following radiation, and a lower-level induction of reactive oxygen species (ROS) after exposure to IR [14]. A separate study has also shown that stem cells derived from breast tumor cell lines have low levels of ROS and possess an anti-oxidant gene expression profile [15]. In addition, Wnt/β-catenin is another mechanism that mediates the radio-resistance of mammary progenitor cells, as suggested by two separate studies performed in murine cells [16, 17]. In glioma, evidence is yet contradictory since stem cells (CD133^+^) have shown radio-resistance by differential regulation of the DNA damage response when exposed to clinically relevant doses of gamma irradiation [18], while others have observed little differences in DNA repair between glioma CD133^+^ cells in larger cohorts of glioma cell lines [11].

We have successfully established protocols for the isolation and maintenance of CSC populations from oral carcinoma cell lines. Further to that, we have shown the existence of a distinct sub-population with a migratory behaviour which is believed to represent invasive tumor cells *in vivo* [19]. This is particularly relevant to therapeutic resistance as this sub-population is also thought to be responsible for the metastatic behaviour of human tumors. In this report, we have examined the response of oral cancer cells to clinically relevant doses of ionising radiation, and have found that oral tumor cells with stem cell properties show enhanced resistance to growth arrest and early apoptotic stimulation as a result of IR treatment. Although all CSC display resistance to IR, those with a mesenchymal expression profile (CD44^hi^/CD24^low^) show a greater level of DNA repair following treatment. By genetic labelling of oral cancer cell lines, we show that mesenchymal CSCs not only comprise the majority of the invasive oral tumor cells, but are also able to resist the DNA damaging effects of ionising radiation in three-dimensional organotypic tissue.

## RESULTS AND DISCUSSION

### Oral CSC are more resistant to IR-induced growth arrest

**Figure 1:**
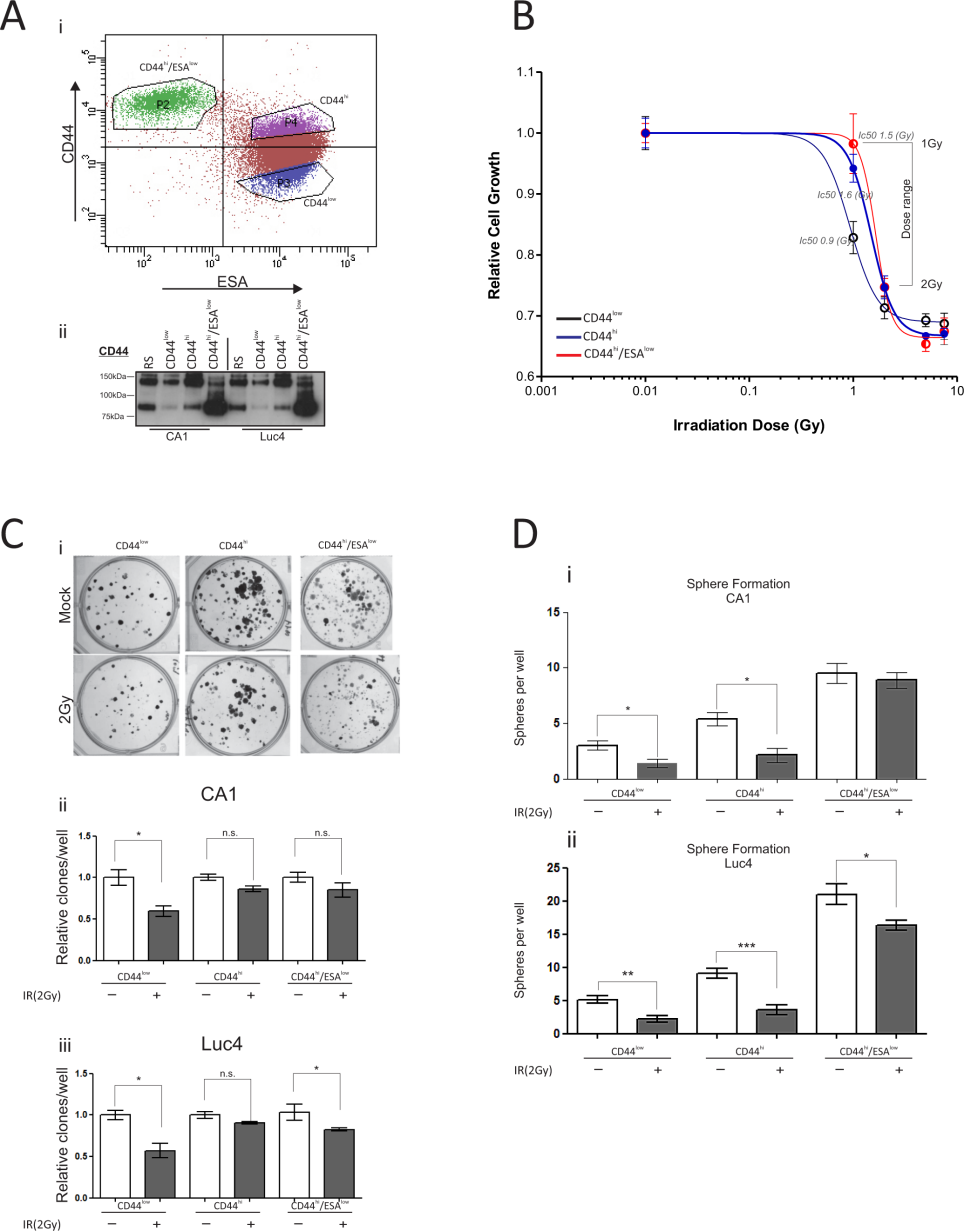
Oral CSC are more resistant to IR-induced growth arrest (**Ai**) Typical flow cytometry profile of CA1 cells stained with CD44-PE/ESA-APC. All three populations CD44^low^/ESA^hi^, CD44^hi^/ESA^hi^, and CD44^hi^/ESA^low^ were flow sorted and grown for five days. (**Aii**) Immuno-blot against anti-CD44, to verify population purity prior to irradiation treatment. (**B**) Epithelial stem cell populations are more resistant to radiation-induced growth arrest. All populations were treated individually, after flow sorting, with varying doses of γ-irradiation and proliferation was measured. CD44^low^/ESA^hi^ was the most sensitive population to growth arrest (Ic50: 1Gy), when compared to the oral CSC populations (CD44^hi^/ESA^low^ Ic50: 1.5Gy; CD44^hi^/ESA^hi^ Ic50: 1.6Gy). (**Ci**) Clonogenic assays were performed to measure the capacity of each population to form colonies after a 10-day period. There is a slightly reduced sensitivity of CSC in response to γ-irradiation, but the differences are not statistically significant (**Cii, Ciii**). (**D**) The sphere forming capacity of CD44^hi^/ESA^low^ cells (motile CSC) remained relatively stable following 2 Gy of IR. Both CD44^hi^/ESA^hi^ and CD44^low^/ESA^hi^ almost completely lost this ability following IR. *P < 0.05, **P < 0.01, ***P < 0.001.

Oral CSC populations can be distinguished by the expression levels of cell surface marker CD44 [8], and sub-categorised to motile or non-motile depending on the levels of ESA expression [19]. To investigate each separate population of oral tumor cells, we first used fluorescence activated cell sorting (FACS) with a combination of anti-CD44 and anti-ESA antibodies to isolate: a) oral CSC, characterized by a CD44^hi^/ESA^hi^ profile, b) oral cancer stem cells undergoing EMT characterized by a CD44^hi^/ESA^low^ profile, c) differentiating oral tumor cells characterized by a CD44^low^/ESA^hi^ profile, and d) oral tumor cells that were sorted at random (RS) (Figure 1Ai). All cells were allowed to grow in culture for five days prior to being exposed to a single dose of ionising radiation. To ascertain that cells retained expression of CD44 throughout culture, each cell fraction was tested for CD44 total protein levels prior to treatment (Figure 1Aii). The CD44 antibody, used for immunoblotting, binds the epitope which is present in the distal region of all CD44 isoforms, therefore recognizing the lower molecular weight CD44 isoform, lacking the variant exons, as well as the other CD44 isoforms (CD44v) of higher molecular weights [19, 25]. CD44^low^ cells express the least amount of CD44, when compared to both CD44^high^ and CD44^high^/ESA^low^ cells, while CD44^high^/ESA^low^ cells express the standard CD44 isoform in much higher levels when compared to CD44^high^ populations, in line with our previous observations [19]. Cells were treated with varying doses of IR and their viability was monitored for the next four days. We found that CD44^low^/ESA^hi^ oral tumor cells, which represent cells with the least self-renewal capacity, were more sensitive to growth arrest (Ic50: 1Gy) compared to the oral CSC populations (CD44^hi^/ESA^low^ Ic50: 1.5Gy; CD44^hi^/ESA^hi^ Ic50: 1.6Gy) (Figure 1B). In addition, for both CA1 and Luc4 HNSCC cell lines, oral CSC had a slight advantage in clonogenic capacity two weeks following a single dose of 2Gy (Figure 1C). The lack of greater difference between those fractions (Figure 1Ci, ii) might be explained by the long culture periods which can allow sufficient time for the self-renewing cells of the CD44^low^/ESA^hi^ fraction to produce equally large colonies. However, we noticed that the sphere forming capacity of CD44^hi^/ESA^low^ cells (motile CSC) remained relatively intact following 2 Gy of IR. In contrast, both CD44^hi^/ESA^hi^ and CD44^low^/ESA^hi^ had a greater reduction in sphere-forming ability following IR treatment (Figure 1Di and ii).

### CSC show preferential activation of DNA damage and repair associated proteins

**Figure 2:**
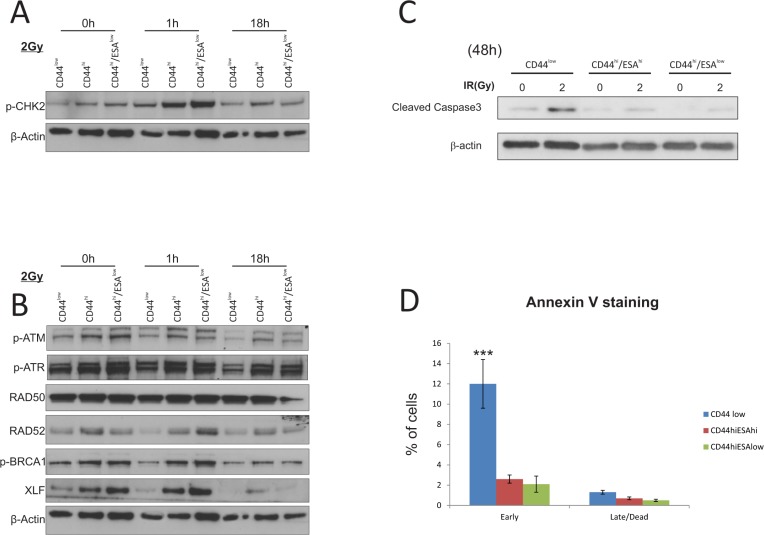
CSC show preferential activation of DNA damage and repair associated proteins Western Blot analysis of protein extracts from CA1 cells. All three populations were flow sorted, then allowed to grow for 5 days prior to a single exposure to 2Gy IR. Cells were allowed to recover prior to being lysed at 1 hour and 18 hours IR. (**A**) Total cell proteins were immuno-blotted against anti-pCHK2, γH2A.x, while β-actin was used as protein loading control. There is preferential activation of both proteins, specifically γH2A.x in CSC populations following IR treatment. (**B**) Total cell proteins were immuno-blotted against anti-pATM, anti-pATR, anti-RAD50, anti-RAD52, anti-pBRCA1, ant-XLF, while β-actin was used as protein loading control. Both types of CSC populations show preferential activation of RAD52, *p*-BRCA1, and XLF suggesting activation of the DNA repair pathway. (**C**) Immuno-blot against anti-cleaved Caspase3 following IR treatment with 2Gy. Longer time points revealed an apoptotic response for CA1 cells, with CSC populations showing a later response to IR treatment. Control CD44^low^/ESA^hi^ cells show dramatic sensitisation to apoptosis starting 72 hours after 1Gy IR, while both CSC populations show a delayed response starting at 48 hours after 5Gy IR. β-actin was used as protein loading control. (**D**) AnnexinV/DAPI staining was performed on all separate fractions 2 days following treatment with 2Gy to detect live (AnnexinV^(−)^/DAPI^(−)^), early apoptotic (AnnexinV^(+)^/DAPI^(−)^), late apoptotic (AnnexinV^(+)^/DAPI^(+)^), and dead (AnnexinV^(−)^/DAPI^(+)^) cells. CD44^low^/ESA^hi^ and CD44^low^/ESA^low^ populations show preferential resistance to early apoptosis when compared to CD44^low^/ESA^low^ control cells. *P < 0.05, ***P < 0.001

Ionising radiation is a strong inducer of both single strand (ss) and double strand (ds) DNA breaks, which in turn lead to activation of cell cycle checkpoints, such as ATM and ATR which regulate downstream checkpoint proteins CHK1 and CHK2 [26–28]. Successful triggering of DNA damage checkpoints leads to the activation of DNA repair, which ultimately determines cell fate. In this respect, we sought to investigate the efficacy of the DNA damage response and repair in sub-populations of HNSCC tumor cell lines. We initially investigated protein levels of different mediators of the DNA damage and repair pathway following irradiation of all sub-populations (Figure 2A, 2B; digital densitometry analysis on Figure S1B), and observed a preferential activation of *p*-CHK2 in CSC populations suggesting an enhanced response to DNA damage, which is often linked to radioresistance. CHK2, as well as other DNA damage response (DDR) proteins are highly expressed in both human embryonic stem cells (ES) and iPS cells, which accords with their proclivity for efficient DNA repair [29]. In stem cell-like enriched populations of nasopharyngeal carcinoma cells, c-MYC positively regulates the expression of CHK1 and CHK2 to confer radioresistance [30], supporting the idea that an enhanced DDR may confer a survival advantage to CD44^hi^/ESA^hi^ and CD44^hi^/ESA^low^ cells. CSC populations also showed preferential activation of RAD52, BRCA1, in the early time point (1 h) following IR, while XLF was consistently upregulated in CSC fractions throughout the irradiation time-course (Figure 2B; densitometry analysis on Figure S1B). It is thought that proliferating cells at the G1 phase of the cell cycle utilise the non-homologous end joining (NHEJ) pathway to repair DNA damage, whereas dividing cells at the G2 phase will utilise the homologous recombination (HRR) pathway for DNA repair [31]. The RAD11-RAD52 epistasis group is responsible for the repair of DSBs in yeast, while overexpression of the human gene can confer γ-ray resistance and induced homologous intra-chromosomal recombination in cultured monkey cells [32]. It is also an alternative mechanism of repair during HRR, in cells with low or deficient expression of BRCA1 and/BRCA2 [33]. More importantly, the elimination of RAD52, by use of peptide aptamer, eliminates cancer stem and progenitor cells in the blood due to excessive accumulation of spontaneous lethal DBs without addition of genotoxic treatment [34]. The elevated levels of XLF and RAD52 prior to IR treatment, or in response to IR, respectively, may suggest that both proteins may be novel targets for oral CSC, the inhibition of which could lead to selective killing of those cells by means of spontaneous DSB induction and/or amplification of DNA damage following IR. Both CSC populations appear to have elevated levels of DDR proteins responsible for both NHEJ (XLF), as well as HRR (RAD52, BRCA1), suggesting that possibly both CSC sub-types are intrinsically capable of utilising either pathway for DNA repair.

Irradiation doses that can cause significant levels of DNA damage, also lead to cell death via apoptosis, due to the presence of extensive unrepaired DNA lesions. We therefore measured the levels of apoptotic markers in all different fractions at various time points following radiation. Although we did not detect any cleavage of PARP or Caspase-9 during the first 18 hours following irradiation (Figure S1), we have seen a sensitisation of CD44^low^ cells 48 h following irradiation with 2Gy. At that time point, CD44^low^/ESA^hi^ and CD44^low^/ESA^low^ cells show significantly reduced levels of cleaved caspase proteins (Figure 2C, digital densitometry shown on Figure S1C). Last, by AnnexinV/DAPI staining (Figure 2D), performed 48 hours after 2Gy IR, we further confirmed the relative apoptotic resistance of both CSC populations, when compared to CD44^low^ control cells.

### CSC have an increased rate of DSB clearance after ionising radiation

**Figure 3:**
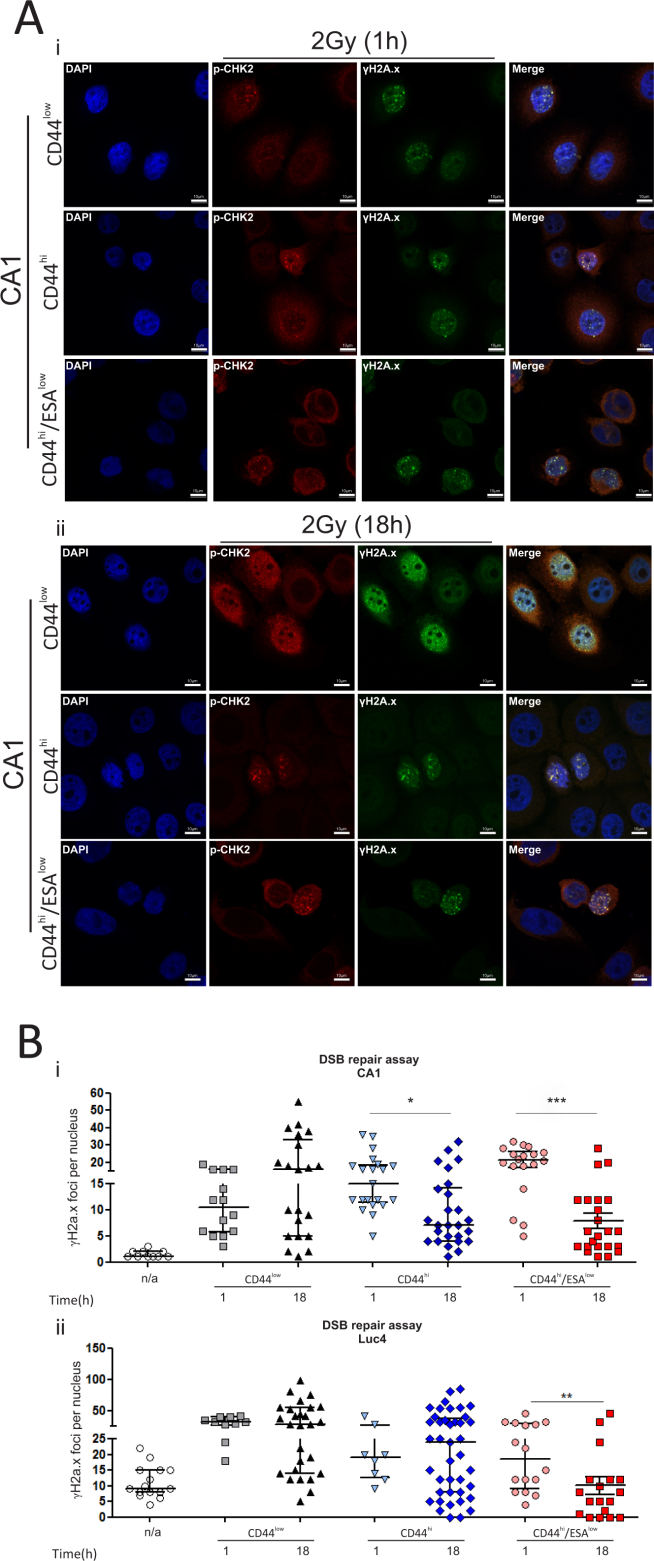
CSC have an increased rate of DSB clearance after ionising radiation Cells were treated with 2Gy IR and immuno-stained to investigate the level of DNA damage and repair following treatment. (**A**) CA1 cells were fixed at set time-points (1 h, 18 h) following treatment. Double strand DNA breaks (DSB foci) were measured by immunofluorescence against anti- γH2A.x and anti-pCHK2. Cells were counterstained with DAPI for nuclear visualisation. DSB foci rapidly accumulated in CA1 cells within an hour after irradiation, regardless of the type of population that was exposed to IR. DNA repair, or DSB foci clearance, was assessed by measuring the remaining γ-H2A.X foci 18 hours post-IR. CD44^hi^/ESA^hi^ and CD44^hi^/ESA^low^ cells displayed a reduced amount of DNA double strand breaks, suggesting quicker and/or more efficient DNA repair. (**B**) DSB foci were counted in each population for both CA1 and Luc4 cells in ImageJ using the cell counter tool. Each data point represents the average amount of nuclear DSB foci (double-positive for γH2A.x and *p*-CHK2) per field. An average of approx. 10 cells was quantified per high power field. Data are representative of two separate experiments performed in two replicate wells each. *P < 0.05, **P < 0.01, ***P < 0.001.

The protein expression profile of CSC suggests a preferential activation of DNA repair proteins that can potentially contribute to an improved rate of DNA repair following IR, hence providing a better chance for survival. Therefore, we tested the rate of DSB clearance from each of those populations by counting the number of γ-H2A.X foci after IR. As previously, cells were flow sorted and treated with low-dose radiation of 1Gy or 2Gy and then examined 1 h and 18 h post-IR for the presence of γ-H2A.X and *p*-CHK2 positive foci (Figure 3Ai, ii). The presence of the phosphorylated form of histone H2A at sites of double strand breaks, is required for the recruitment of proteins essential for the process of DNA repair [35, 36]. As is evident from the images, DSB foci accumulate in both CA1 and Luc4 cells within an hour after irradiation. The rate of DNA repair, or the rate of reduction in the number of DSB foci, can be assessed by measuring the remaining γ-H2A.X foci several hours after treatment. We had already seen that the 18 h time-point is too early for apoptotic stimulation to result from radiation treatment (Figure 2C), excluding the possibility that cells with high levels of DNA damage foci might be lost by death. By counting the remaining double strand breaks in all different sub-populations we find that CD44^hi^/ESA^hi^ and CD44^hi^/ESA^low^ cells displayed a reduced amount of DNA double strand breaks, suggesting quicker and/or more efficient DNA repair (Figure 3Bi, ii). In line with our observations, Dao et al. has also proposed that glioblastoma stem cells (CD133^+^) display accelerated DNA repair [18]. These observations suggest that the two subpopulations of CSC are more capable of responding to irradiation-induced double strand breaks and could potentially be more capable of repopulating a tumor following treatment by ionising radiation.

### CD44^hi^/ESA^low^ is the invasive population of oral tumor cells

**Figure 4:**
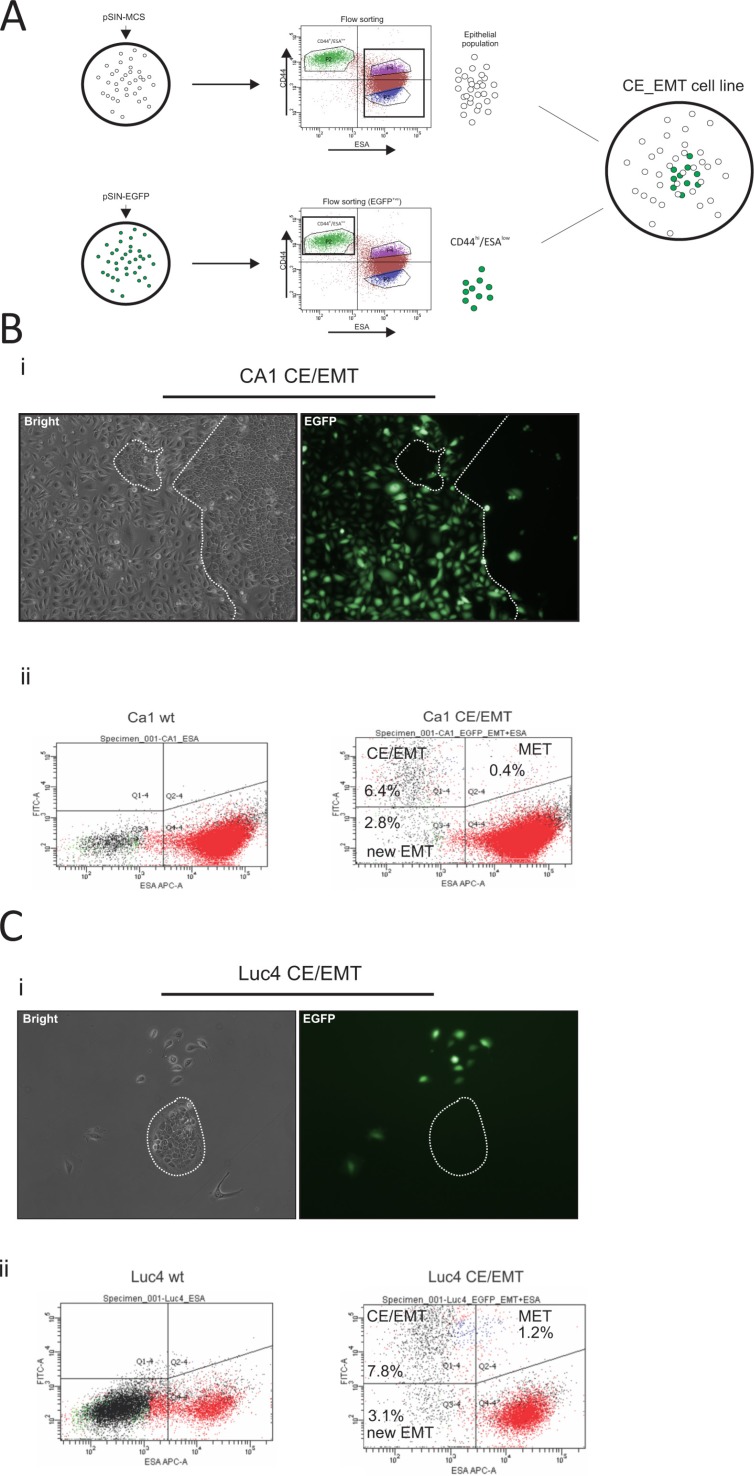
Generation and characterisation of EMT-CE cell lines CA1 and Luc4 parental cell lines were transduced with either pSIN-MCS or pSIN-EGFP retroviral vectors (**A**). pSIN-MCS transduced cells were completely depleted of CD44^hi^/ESA^low^ population, while the pSIN-EGFP transduced cell lines were flow sorted and only CD44^hi^/ESA^low^ were isolated. Both types were mixed at a ratio which mimicked that of the parental wild-type cell line. (**B, C**) Cells were later grown for up to 14 days. By 7 days of culture, the very distinct phenotype of mesenchyme-like (CD44^hi^/ESA^low^) cells was almost always accompanied by clear expression of EGFP marker protein (**Bi, Ci**) whereas epithelial cells did not display expression of EGFP. By 14 days of culture, a minority of EGFP^(+)^ cells with epithelial morphology as well as a significant number of EGFP^(−)^ cells with a mesenchymal phenotype became evident. After prolonged culture we were able to visualise the dynamics of epithelial to mesenchyme transition (EMT) by tracing the EGFP^(−)^ CD44^hi^/ESA^low^ cells (**Bii, Cii**) as well as of mesenchyme to epithelial transition (MET) by tracing the EGFP^(+)^ CD44^hi^/ESA^hi^ cells. The MET rate appeared to be higher for Luc4 cells compared to CA1 cells, while the rate of EMT seemed to be similar to both types of cells. Continuous monitoring of both reconstituted cell lines showed that a part of the original CD44^hi^/ESA^low^ population remained permanently at a mesenchymal state while the remaining population was recycled within the parental cell line.

Although cancer stem-like cells with a mesenchyme expression profile are radio-resistant when cultured independently of other cells, *in vivo* tumors possess a mixture of cells permitting interactions between the sub-populations of cancer cells and the stromal cells. We therefore decided to maintain the proportion of the parental cell lines, but in such a way that CD44^hi^/ESA^low^ cells could be easily tracked. CA1 and Luc4 lines were stably transduced with either pSIN-MCS (empty control) or pSIN-EGFP retroviral vectors (Figure 4A). The pSIN-MCS transduced cells were sorted to completely deplete them of the CD44^hi^/ESA^low^ population, while the pSIN-EGFP transduced cell lines were flow sorted to isolate only CD44^hi^/ESA^low^ cells (Figure 4A). Finally, both types were mixed at a ratio mimicking that of the parental cell line. To establish the stability of our system, we let the cells grow in culture for up to 14 days. By 7 days of culture, the very distinct phenotype of mesenchyme-like elongated cells (CD44^hi^/ESA^low^) cells was almost always accompanied by clear expression of EGFP marker protein (Figure 4Bi, Figure 4Ci) whereas epithelial cells did not display expression of EGFP. Cells with an epithelial stem-like morphology, small round cells with high nuclear/cytoplasmic ratio, and organised into tight compact colonies termed holoclones [37, 38], were almost always seen as EGFP^(−)^ (clear depiction of a holoclone EGFP^(−)^ shown in Figure 4Ci).

By 14 days of culture we noticed a minority of EGFP^(+)^ cells with epithelial morphology as well as a significant number of EGFP^(−)^ cells with a mesenchymal phenotype. Interestingly, using the prolonged 14 day culture we were able to visualise the dynamics of epithelial to mesenchymal transition (EMT) by tracing the EGFP^(−)^ CD44^hi^/ESA^low^cells (2.8% new EMT ratio Figure 4Bii left; population Q3–4) as well as of mesenchyme to epithelial transition (MET) by tracing the EGFP^(+)^ CD44^hi^/ESA^hi^ (0.4% Figure 4Bii right; population Q2–4) cells. The MET rate appeared to be higher for Luc4 (1.2% Figure 4Cii right; population Q2–4) cells compared to CA1 cells, while the rate of EMT seemed to be similar to both types of cells. Continuous monitoring of both reconstituted cell lines showed that a part of the original CD44^hi^/ESA^low^ population remained permanently at a mesenchymal state while the remaining population was recycled within the parental cell line, in agreement with our previous work showing the presence of uni-potent and bi-potent CD44^hi^/ESA^low^ cells [19].

**Figure 5:**
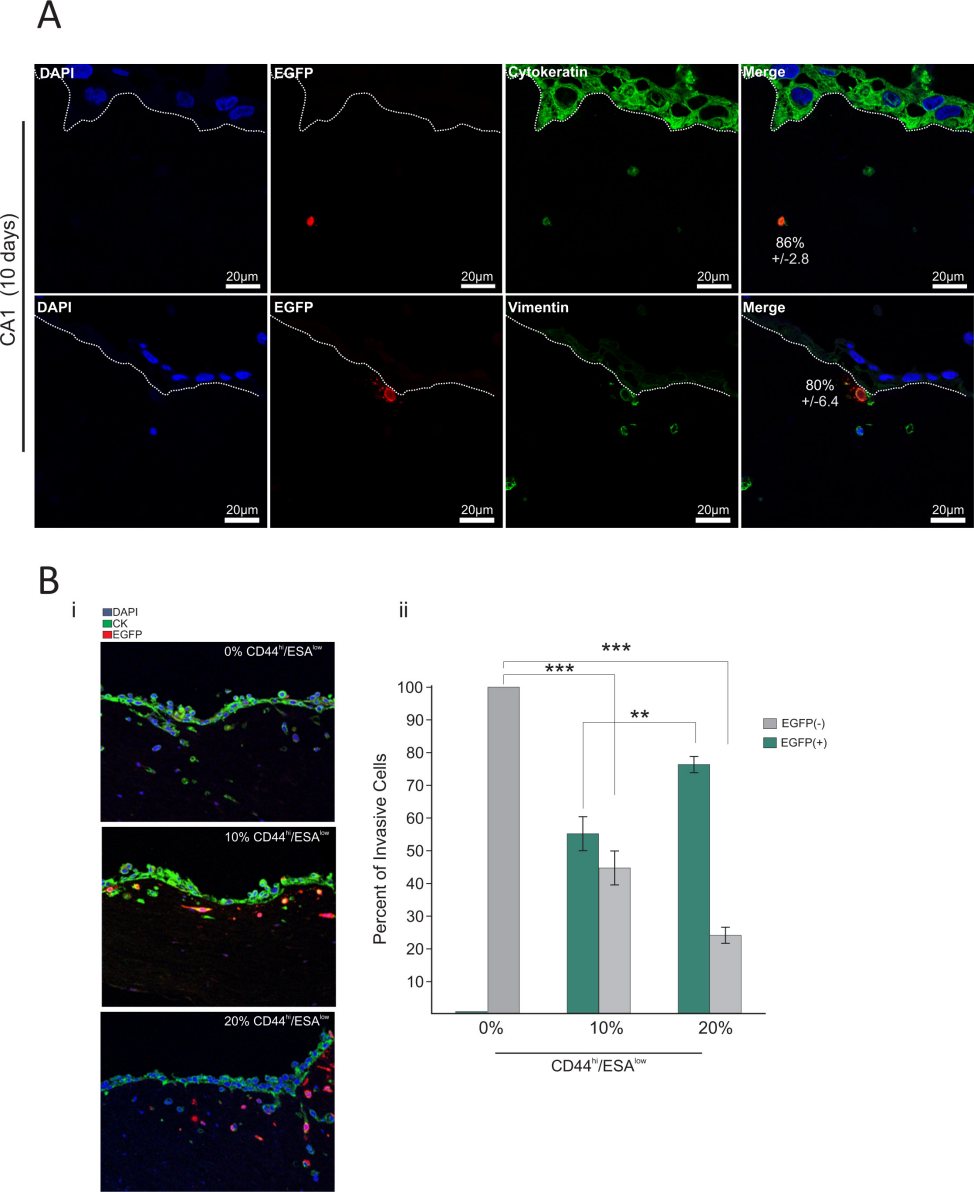
CD44^hi^/ESA^low^ is the invasive population of oral tumor cells The EMT-CE cells derived from the CA1 line were cultured in 3D collagen:matrigel gels for a total period of 10 days. (**A**) Gels were immuno-stained with anti-EGFP, anti-Cytokeratin, and anti-Vimentin. DAPI was used as a counterstain to visualise the nucleus. A large majority (86% ± 2.6%) of EGFP^(+)^ CA1 cells was able to invade through the stroma of organotypic cultures. Furthermore, EGFP^(+)^ cells comprised the majority of all invasive cells for both CA1 and Luc4 cell lines. 80% ± 6.4% of invasive EGFP^(+)^ was also positive for Vimentin. (**B**) EMT-CE CA1 line was re-constituted with varying percentages of EMT cells (0%, 10%, and 20% CD44^hi^/ESA^low^) and cultured for 14 days on top of 3D collagen:matrigel organotypic cultures. CA1 parental line without any CD44^hi^/ESA^low^ cells showed invasion driven by tumor cells that have undergone EMT while growing on the organotypic tissue (EGFP^−^). The invasion index is not further increased regardless of the amount of CD44^hi^/ESA^low^ cells that are added to the parental cell line (**Bi, Bii**). In the 10% variant the majority of invading cells are EGFP^(+)^, while in the 20% variant of the CA1 line, almost all invading cells were EGFP^(+)^, suggesting that only very few cells invaded into the organotypic tissue as a result of new EMT. *P < 0.05, **P < 0.01, ***P <0.001.

We then investigated the behaviour of the CD44^hi^/ESA^low^ sub-type in physiologic 3D culture conditions that mimic *in vivo* tissue architecture. Therefore, we used a collagen/matrigel organotypic matrix oral epithelia to culture the CA1_EGFP-EMT and Luc4_EGFP-EMT cell lines for a period of 10 days in the presence of fibroblasts which comprised the ‘stroma’. We found that 86% ± 2.8% of EGFP^(+)^ cells in the CA1 line, and 92% ± 9.1% of EGFP^(+)^ cells in the Luc4 line were able to invade through the stroma of these organotypic cultures (Figure 5A). Furthermore, those cells comprised the majority of all invasive cells for both cell lines providing, for the first time to our knowledge, visual evidence that CD44^hi^/ESA^low^ cells is the invasive population of oral tumor cells. From our 2D culture observations, we show that the majority of EGFP^(+)^ cells are maintained in a mesenchymal state characterised by CD44^hi^/ESA^low^ profile even after 14 days in culture. Similarly, in 3D organotypic tissue, we find that genetically modified CD44^hi^/ESA^low^ cells (EGFP^(+)^) also remain Vimentin^+^ by 80% ± 6.4% within the 14 days of culture (Figure 5A (lower panel) and Figure S3). As a result, we propose that by selecting CD44^hi^/ESA^low^ populations from oral tumor cell lines, we can identify the population of cells responsible for tumor invasion in both cell lines. The remaining Vimentin^+^cells comprised epithelial cells that had undergone EMT (Vimentin^+^/EGFP^−^; Figure S3) during the course of tissue formation, as well as stromal fibroblasts. This agrees with our observations (Figure 4Aiii; population Q3–4) showing that new EMT becomes evident by the 14^th^ day of culture.

We further tested this by producing lines with a) 0% CD44^hi^/ESA^low^ and b) 10% of CD44^hi^/ESA^low^ and c) 20% of CD44^hi^/ESA^low^ and have cultured each for 14 days in organotypic cultures. In this system, the rate of EMT can be indirectly measured by the amount of invading cells that are EGFP^(−)^. The CA1 parental line without any CD44^hi^/ESA^low^ cells showed remarkable invasion ability, driven by tumor cells (EGFP^(−)^) that have undergone EMT during that period. Surprisingly, this invasion index was not further increased regardless of the amount of CD44^hi^/ESA^low^ cells that were injected into the parental cell line (Figure 5Bi, ii). However, in the 10% variant, the majority of invading cells were EGFP^(+)^, while in the 20% variant of the CA1 line, almost all invading cells were EGFP^(+)^, suggesting that only very few cells invaded into the organotypic tissue as a result of new EMT (Figure 5Bii). Our results show that the rate of EMT is reduced and/or inhibited in the presence of an established CD44^hi^/ESA^low^ population and therefore, suggesting a feedback mechanism that maintains an equilibrium between CD44^hi^/ESA^low^ and CD44^hi^/ESA^hi^ populations and results in a stable invasion index in all cases. This indicates that even with complete eradication of CD44^hi^/ESA^low^ cells, the invasive tumor sub-populations can be replenished quickly from the established tumor epithelium. In the case of many carcinomas, EMT is induced, or assisted, by growth factor signals originating from tumor associated stroma (i.e. PDGF, TGF-β, EGF, HGF) [39–41], or even by the deposition of ECM proteins like fibronectin, as is the case in mammary epithelium [7]. It would be interesting to know the involvement of varying stromal cells in the EMT process but it appears that combined treatments, including both selective eradication of CD44^hi^/ESA^low^ cells as well as secondary blockade of the EMT process may be necessary to inhibit tumor invasion and subsequent metastasis. Interestingly the remaining small fraction of EGFP^(+)^ cells were found in the upper layers of the re-constituted epithelium suggest that MET is occurring during 3D culture, which is consistent with our observations on 2D cultures (Figure 4Bii and Figure 4Cii) where MET had occurred during a 14-day period.

### Invading tumor cells are radio-resistant in organotypic cultures

**Figure 6:**
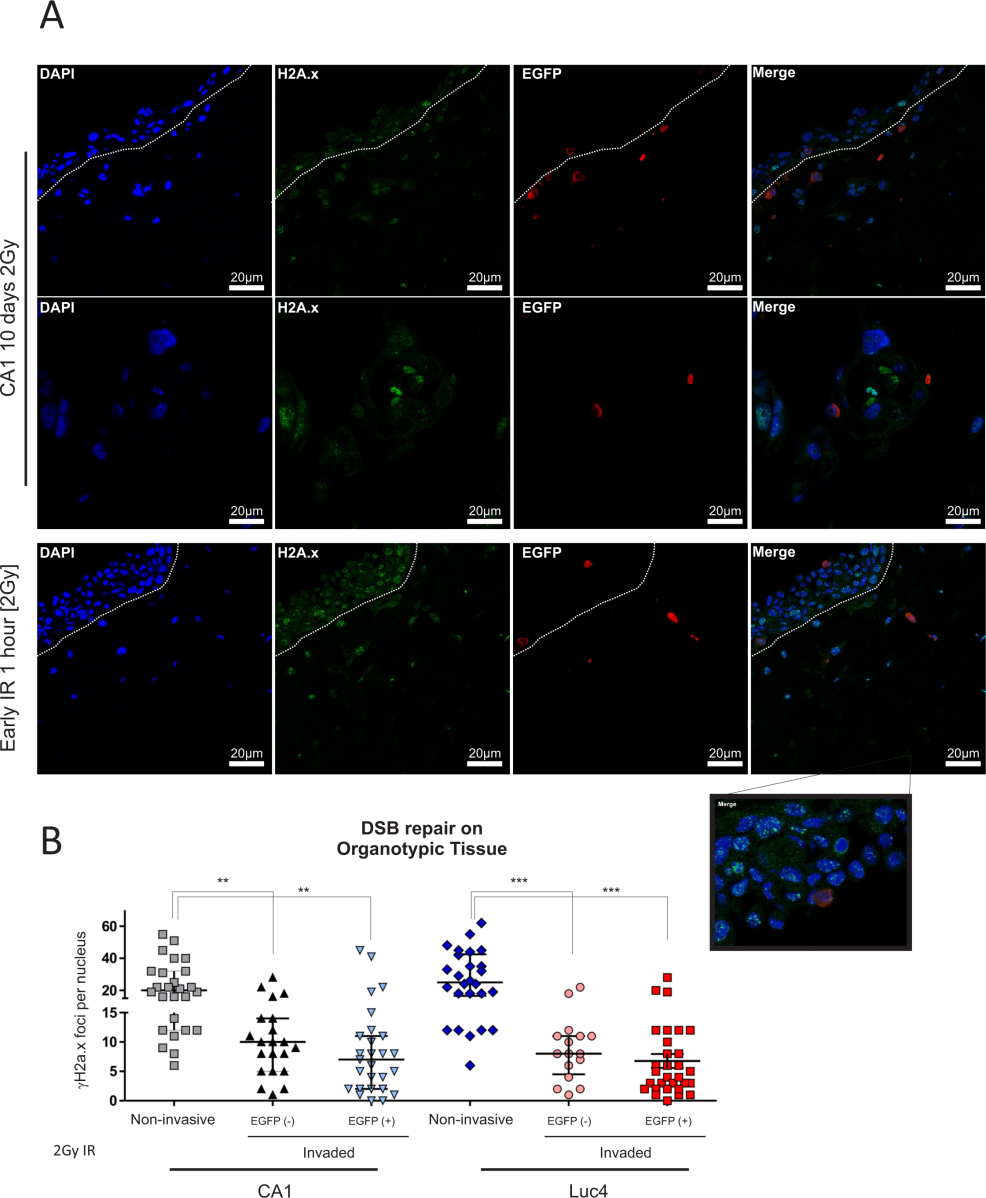
Invading tumor cells are radio-resistant in organotypic cultures To assess the differential response of each population to IR treatment in a physiological 3D environment, EMT-CE lines were grown on top of collagen:matrigel (3:1 ratio) gels for 10 days prior to being exposed to a single dose of 2Gy IR. Gels were allowed to grow for 1 hour and 3days post-irradiation. (**A**) The tissues were stained with anti-EGFP and anti- γH2A.x and DSB foci were counted in each condition. (**B**) Each data point represents the average amount of nuclear DSB foci (positive for γH2A.x) per field. An average of approx. 10 cells was quantified per high power field. Data are representative of two separate experiments performed in two replicate wells each. All invasive cells, regardless of their prior status (EMT or epithelial), showed enhanced DSB clearance 3 days following IR treatment with 2Gy. *P < 0.05, **P < 0.01, ***P < 0.001.

As we were able to identify and track the EMT population of CSC in 3D organotypic culture, we wanted to examine its behaviour in response to IR in a physiological 3D environment. We cultured CA1_EGFP-EMT cells as 3D organotypic tissues for 10 days to allow a malignant epithelium to be established. Then, we exposed the tissues to a single IR dose of 2Gy and, to examine the effect of IR treatment, allowed the cells to return to normal conditions for 1 hour or 72 hours. 2Gy irradiation induced DSB within 1 hour, a rate similar to that observed in 2D cultures (Figure 6A, 6B). Three days following irradiation treatment, we observed that all invaded cells had been able to clear DSB more efficiently when compared to all other cells in the 3D epithelium (Figure 6B). We did not see significant differences between the amounts of DSB remaining in EGFP^−^ (new EMT CSC) or EGFP^+^ (pre-established EMT CSC) cells. Taken together, these data support our notion that the CD44^hi^/ESA^low^ population is the invasive population of tumor cells in 3D tissue and, further to that, also displays enhanced DNA repair activity. In a recent report it was shown that non-small cell lung carcinoma cells that survive ionizing radiation, have a complex phenotype which included properties of CSC and EMT markers [42], which supports the notion that CSC populations, and specifically those cells with EMT traits, can show relative resistance to the effects of IR. Interestingly, we also observed that IR treatment in 2D cultures stimulated the transition of EMT CE cells towards a more epithelial phenotype, suggesting the induction of MET (Figure S2), while increasing the IR dose past 2Gy made little difference to this effect. The same dose of IR (2Gy) can also alter the metastatic and migratory ability of breast tumor cells in a time-dependent manner [43]. Those observations could be of importance since, in combination with the radio-resistance of EMT cells in a 3D environment, IR treatment may influence the capability of resistant EMT cells to be re-established (by re-epithelisation) at local metastatic sites within the regions affected by IR treatment.

We have shown that a defined CD44^hi^/ESA^low^ population of head and neck tumour cells is capable of invasion into 3D organotypic tissues, and that this population is resistant to therapeutically relevant doses of IR. This can be attributed to enhanced clearance of IR-induced DNA damage, possibly due to up-regulation of certain DDR proteins, some of which could potentially also serve as novel targets for the sensitisation of CSC to IR treatment. The presence of the motile CD44^hi^/ESA^low^ population is not a pre-requisite for cell invasion as invasion (and possibly metastasis) could still occur via the observed generation of *de novo* EMT which led to the quick re-establishment of the motile CD44^hi^/ESA^low^ fraction. It is possible that sensitization of CD44^hi^/ESA^low^ cells to IR, followed by secondary EMT blockade could be more beneficial in inhibiting cell invasion and subsequent metastasis.

## MATERIALS AND METHODS

### Cell culture

Primary HNSCC derived cell lines [19]: CA1, Luc9, and Luc4 were maintained in FAD medium consisting of DMEM supplemented with 25% Ham's F12 medium (Gibco, Invitrogen), 10% foetal calf serum (FCS), 1% penicillin/streptomycin (Invitrogen, Paisley, UK), and various mitogens (0.4 μg/ml hydrocortisone, 0.1 nM cholera toxin, 5 μg/ml transferrin, 20 pM lyothryonine, 0.18 mM adenine, 5 μg/ml insulin and 10 ng/ml EGF). All cells were grown at 37°C in a humidified atmosphere of 10% (DMEM/RM+) or 5% (K-SFM, EpiLife) CO_2_/95% air.

### Organotypic cultures

Collagen and matrigel were mixed at a ratio of 5.25 volumes (collagen type I) and 1.75 (Matrigel), with 1 volume of 10 × DMEM, 1 volume FBS, and 1 volume of stromal cell suspension (5 × 10^5^ human foetal fibroblasts, or normal oral fibroblasts). The mixture was added into wells of a 24-well plate (1 ml/well) pre-coated with diluted collagen type I for 1 hr at 37°C in a humidified atmosphere of 10% (1:100 in PBS). The mixture was left to set for 1 h at 37°C in a humidified atmosphere of 10%. Next, 1 ml of the cancer cell suspension (5 × 10^5^ cancer cells per ml) was added on top of the gels and left 1 h at 37°C in a humidified atmosphere of 10%. Next day, gels were raised at the air liquid interface, by placing on top of metal grids. Experiments were carried out in triplicate, and medium was changed on alternate days. Gels were harvested at indicated time points by fixation in 4% formaldehyde overnight at room temperature. Gels were then briefly washed in 1x PBS, bisected and put in 70% ethanol. Finally, gel organotypic tissue was paraffin-embedded and processed for immunohistochemistry at the Pathology Core Facility of Blizard Institute of Cell and Molecular Science (Barts and The London School of Medicine and Dentistry). Cells were defined as being invasive and/or invaded, when they were detected (double positive for anti-pan-Cytokeratin and DAPI) below the collagen/epithelial interface of the organotypic gel. Both cell lines (CA1, Luc4) used in this study formed a relatively uniform basal layer of the re-constructed epithelium. Immnohistochemistry with anti-Pan-Cytokeratin allows the visualisation of the epithelial architecture, and thus the lower part of the basal epithelial layer could be easily identified.

### Plasmid construction

The retroviral vector pSIN-IP-GFP (Self-Inactivating-Internal Promoter-Green Fluorescence Protein [20] vector kindly provided by Dr. Paul Khavari (Stanford University School of Medicine, California, USA). In some experiments, the pSIN-MCS (MCS) empty viral vector bearing no transgene was used as an additional control. Transduction of MCS or EGFP did not produce any detectable biological artefacts in any of the experiments.

### Clonogenic assays and cell counting

Keratinocytes were counted using CAsy^®^ Cell Counter (Innovatis). Each sample was prepared three times in CAsyTon^®^ (Innovatis) buffer and triplicate measurements of 200 μl sample volume were taken each time. Population doublings were calculated as follows: *PD* = 3.32 * [log_10_ (N1) – log_10_ (N0)] where N1 = total yield and N0 = initial number of seeded keratinocytes. Clonogenicity percentages were calculated as follows: Clonogenicity % = VC × 100/N0 where VC = visible colonies and N0 = initial number of keratinocytes. For colony forming assays, keratinocyte cultures were washed once with PBS and were fixed in 4% (v/v) formaldehyde in PBS for 20 minutes at room temperature. Cells were washed 1x with PBS and stained with 1% Rhodamine B (Sigma, Dorset, UK) in PBS for 30 minutes at room temperature. Colony area coverage measurements were performed using Adobe Photoshop CS4 and CorelDraw X by using the color selection tool to acquire quantitative pixel counts. Clonogenic assays were carried out in 6 replicate wells for each sample.

### Fluorescence activated cell sorting

Fluorescence activated cell sorting experiments were performed on BD FACSAria Cell-Sorter (BD Biosciences) at Blizard Flow Cytometry core facility by Dr. Gary Warnes. For fluorescence activated cell sorting, cells were tyrpsinised and washed once with cold PBS and then re-suspended in cold FACS buffer consisting of PBS with 5% foetal bovine serum (FirstLink), and 1% penicillin/streptomycin (Gibco Invitrogen). Cells were then stained by direct immunofluorescence for 15 minutes on ice with either PE conjugated anti-CD44 β1 (BD Biosciences), APC conjugated anti-ESA (BD Biosciences) or both at a concentration of 10 μl/10^6^ cells in 100 μl of FACS buffer. Cells were spun at low speed centrifugation at 140x g for 3 minutes, washed once in cold PBS, spun again at 140x g for 3 minutes, and finally re-suspended in cold FACS buffer containing 200 ng/ml DAPI (DAPI dilactate – Sigma) for dead cell exclusion during analysis. DAPI positive cells were gated out and fractions were sorted by selecting the highest/lowest 20% of CD44-stained cells or high/low populations stained with anti-ESA antibody. Control sorted populations were selected by sorting randomly from the total pool of DAPI negative cells. Keratinocytes were then plated through the automated cell dispenser to achieve maximum accuracy, directly on 6-well plates at concentrations ranging from of 54 – 1×10^3^/cm^2^ for clonogenic assays, or as required.

### Retroviral transduction

Phoenix packaging (PhxA) cells were plated in 10 cm dishes and were transfected with 10 μg of DNA (pSIN-EGFP, pSIN-MCS (emptly vector containing the multiple cloning site), using FuGENE 6 reagent (Roche) according to manufacturer's instructions. Transfected Phoenix cells were incubated for 48 hrs and then sub-cultured in selection medium with puromycin (3 ug/ml) (Sigma Aldrich). Cells were maintained in selection medium for 1 week. Target cells were pre-incubated for 5 minutes in growth medium containing 5 μg/ml polybrene (hexadimethrine bromide; Sigma) before replacing with retroviral supernatant containing the same concentration of polybrene to facilitate infection. The cells in culture dishes with viral supernatant were then centrifuged (350x g) at 32°C for 1 hour before retroviral supernatant was replaced with normal growth medium and kept in normal culture condition.

### Immunoblotting

Protein extraction and separation on SDS-PAGE gels and immunoblotting was performed as previously described (5). The isolation and irradiation of separate populations from CA1 and Luc4 cell lines was repeated twice. Each set of samples (two sets) was immunoblotted twice. Primary antibodies used were rabbit anti-phospho-CHK2 (Thr68) (Cell Signaling), rabbit anti-phospho-ATM (Ser1981) (Cell Signaling), rabbit anti-phospho-ATR (Ser428) (Cell Signaling), rabbit anti-RAD50 (Cell Signaling), rabbit anti-RAD52 (Cell Signaling), rabbit anti-phospho-BRCA1 (Ser1524), rabbit anti-XLF (Cell Signaling), and mouse anti-β-Actin (Sigma). Secondary antibodies used were polyclonal rabbit anti-mouse immunoglobulin/HRP (DakoCytomation), and polyclonal goat anti-rabbit immunoglobulin/HRP (DakoCytomation). Densitometry was performed on scanned immunoblot images using the ImageJ gel analysis tool [21]. The gel analysis tool was used to obtain the absolute intensity for each experimental protein band and its corresponding protein loading (β-Actin) control band.

### Annexin V staining

Annexin V staining on isolated fractions of oral cancer cells, following irradiation, was performed as previously described [22].

### Immunohistochemistry, fluorescence microscopy and digital pixel densitometry

Organotypic sections were initially heated at 60°C for eight minutes. Sections were then deparaffinised in Xylene (3x for 5 minutes) and then dehydrated in (95% ethanol, 90% ethanol, 70% ethanol for 5 minutes each) and finally rehydrated by incubating for 5 minutes in water. Antigen unmasking was performed by incubating sections in sub-boiling temperature for 20 minutes in high pH (9.0) antigen retrieval solution (DakoCytomation). The sections were allowed to cool to room temperature for 30 minutes before washing 2x in TBS 0.025% tween-20 (Sigma) and were then permeabilised with TBS 0.2% Triton-X (Sigma) for 10 minutes at room temperature. Protein blocking was performed by 1 hour incubation in 10% Goat Serum (Sigma) in TBS (0.025% Tween-20) at room temperature. Primary antibody probing was performed by overnight incubation at 4°C in 1%BSA TBS (0.025% Tween-20). Sections were then washed 3x in TBS (0.025% Tween-20) and probed with fluorescence conjugated secondary antibodies (diluted in 1% BSA in TBS 0.025% Tween-20) for 1 hour at room temperature. Finally sections were washed 3x in TBS 0.025% Tween-20 and mounted with Shadon Immu-Mount^TM^ (Thermo Scientific) solution cotaining DAPI (DAPI dilactate – Sigma) at a final concentration of 300 ng/ml. For frozen section staining, the fixation protocols included 4% (vol/vol) formaldehyde in PBS for 20 minutes at room temperature or ice cold methanol: acetone (1:1) for 10 minutes at room temperature. The staining procedure was thereafter the same as mentioned above for paraffin embedded tissues. Antibodies used for immune-fluorescence were mouse anti-γH2A.Xantibody (clone JBW301K;Millipore), rabbit anti-phosho-CHK2 (Cell Signaling), rabbit monoclonal anti-EGFP (Cell Signaling), mouse anti-Vimentin (Dako) on paraffin sections as described [23]. The reaction product was visualised with Alexa Fluor 488 goat anti-rabbit IgG, and 546 secondary antibodies (Molecular Probles, Invitrogen, Paisley, UK) for fluorescence microscopy imaging in cultured cells. Whilst we have also used ImageJ program for pixel densitometry, foci and cell counting and obtained similar results, Photoshop was the preferred choice for its accuracy, reproducibility and ease of use.

### Cell viability and anchorage-independent cell transformation assay

CellTiter-GloTM luminescent assay (Promega, Madison, WI, USA) was performed according to manufacturer's instruction to quantify ATP levels of metabolically active adherent cells. Sphere formation assays were performed as described previously [24].

## SUPPLEMENTARY FIGURES


