# Supplementary material for: Invasive oral cancer stem cells display resistance to ionising radiation
Source: Oncotarget. 2015 Nov 2;6(41):43964–77. doi: 10.18632/oncotarget.6268 (PMC4791279; doi:10.18632/oncotarget.6268)
Supplement: Supplementary file 1 [file oncotarget-06-43964-s001.pdf]

Invasive oral cancer stem cells display resistance to ionising radiation

Supplementary Figures

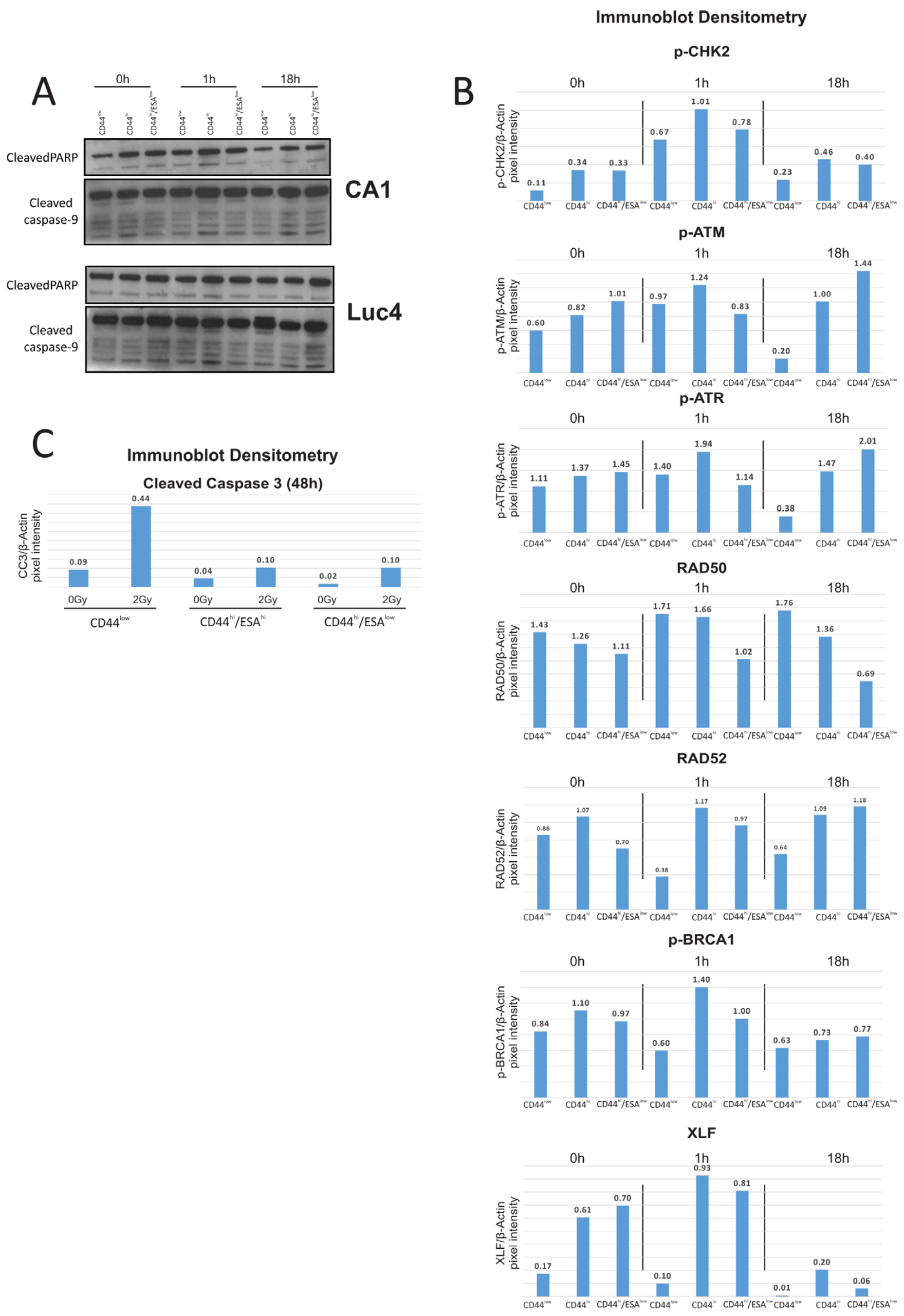

Supplementary Figure S1: Early apoptotic response of CSC. **(A)** Total cell proteins were immuno-blotted against anti-cleaved PARP and anti-cleaved caspase-9. There is lack of apoptotic activation for all treatment within the first 18 hours following IR treatment. Digital densitometry analysis of the immunoblots shown in Figure 2A and 2B is shown on panel **(B)**, while panel **(C)** shows digital densitometry analysis of immunoblots shown in Figure 2C, quantifying the absolute intensity of protein bands/absolute intensity of the protein loading control (β-Actin) in oral CSC.

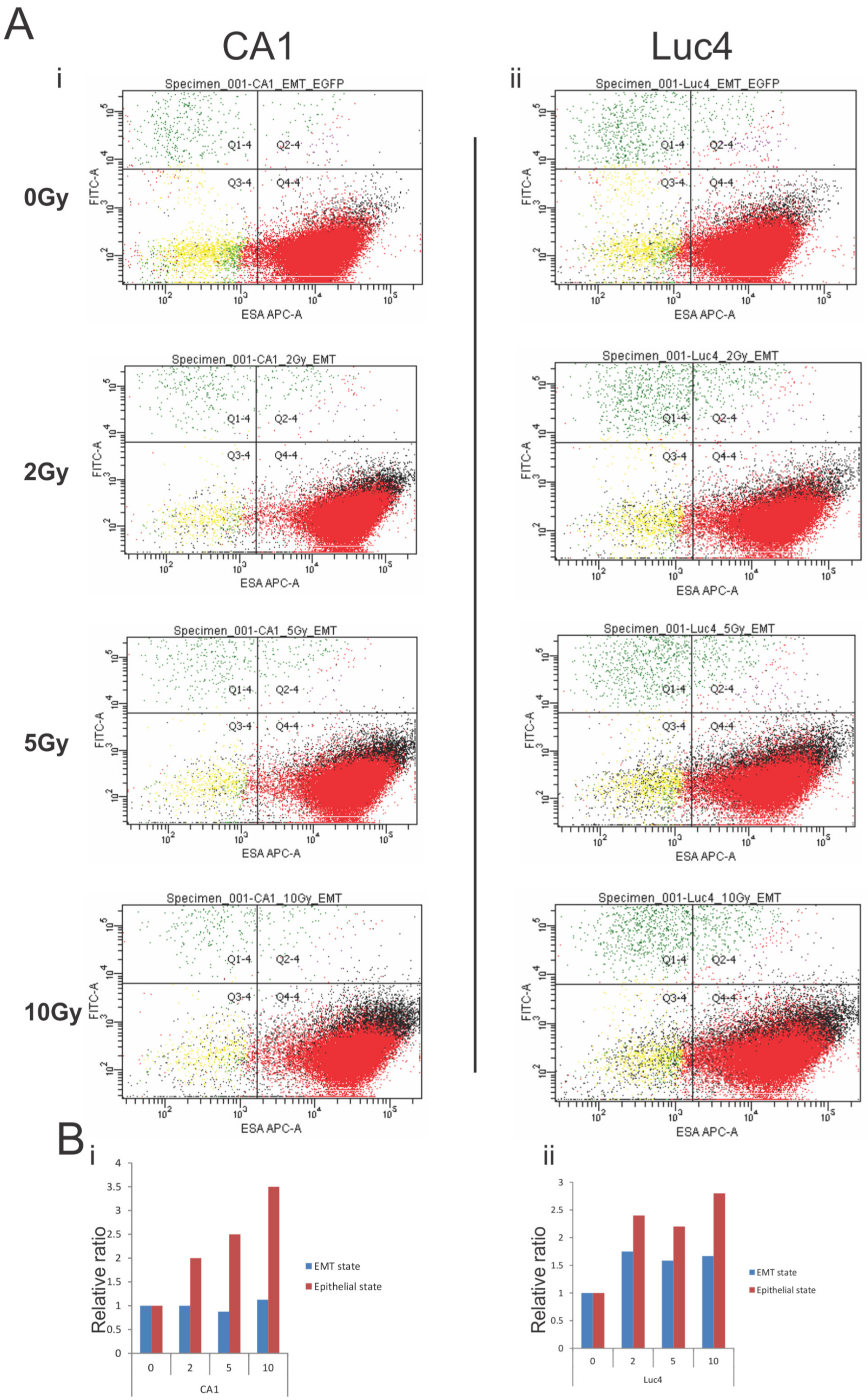

Supplementary Figure S2: Response of the EMT CE lines to IR. (**A**) Cells were exposed to various doses of IR and were let grow for 3 days prior to being analysed by flow cytometry for ESA expression. CA1 (**ii**) EMT cells are converting to an epithelial-like state (MET) with increasing doses of radiation. Within the physiological range of 2Gy, cells are converting to the epithelial phenotype by a ratio of 1:2. Luc4 (**ii**) cells also seem to undergo MET with increasing doses of IR albeit at lower levels ratio level when compared to that of CA1 cells.

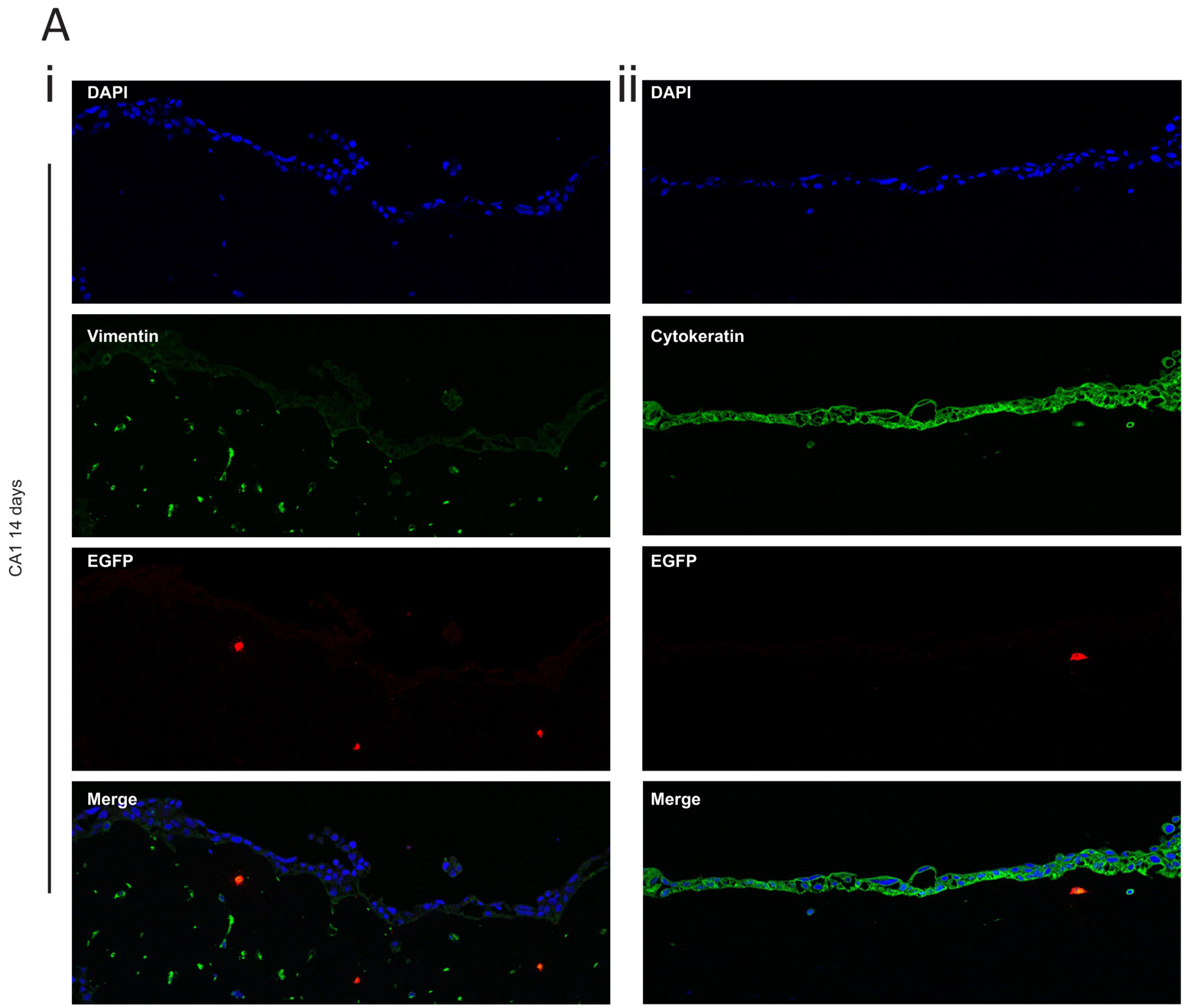

Supplementary Figure S3: CD44^{hi}/ESA^{low} retain Vimentin expression in organotypic cultures. (**A**) The EMT-CE cells derived from the CA1 line were cultured in 3D collagen:matrigel gels for a total period of 14 days. Gels were immno-stained with (**i**) anti-EGFP plus anti-Vimentin or with (**ii**) anti-EGFP and anti-Cytokeratin. DAPI was used as a counterstain to visualise the nucleus. Almost every cell below the epithelium was marked as Vimentin positive but very few cells were of epithelial origin, as judged by cytokeratin staining.
